# Suicidal expressions in young Swedish Sami, a cross-sectional study

**DOI:** 10.3402/ijch.v72i0.19862

**Published:** 2013-01-17

**Authors:** Lotta Omma, Mikael Sandlund, Lars Jacobsson

**Affiliations:** 1 Department of Child and Adolescent Psychiatry, Gällivare Hospital, Gällivare, Sweden; 2 Department of Clinical Sciences, Division of Psychiatry, Umeå University, Umeå, Sweden

**Keywords:** suicide ideation, suicide attempts, gender, reindeer herders, indigenous Sami, attitudes toward suicide

## Abstract

**Objectives:**

To investigate the experience of suicidal expressions (death wishes, life weariness, ideation, plans and attempts) in young Swedish Sami, their attitudes toward suicide (ATTS), and experience of suicidal expressions and completed suicide in significant others and to compare with Swedes in general.

**Methods:**

A cross-sectional study comprising 516 Swedish Sami, 18–28 years of age together with an age and geographically matched reference group (*n*=218). Parts of the ATTS questionnaire have been used to cover different aspects of the suicidal complex.

Data were analysed with regard to gender, occupation, counties and experience of negative societal treatment due to Sami background.

**Results:**

Both young Sami and young Swedes reported suicidal ideation, life weariness, and death wishes in a high degree (30–50%), but it was more common among the Sami. Having had plans to commit suicide showed a significant gender difference only in the Sami. The prevalence of suicide attempts did not differ significantly between Sami and Swedes. Subgroups of the Sami reported a higher degree of suicidal behaviour, Sami women and reindeer herders reported a 3, 5-fold higher odds of *suicide attempts* and a 2-fold higher odds *having had plans committing suicide*. Sami living in Vasterbotten/Jamtland/Vasternorrland and Sami with experience of ethnicity related bad treatment 2-fold higher odds of suicidal plans compared to those living in other counties.

**Conclusion:**

An increased occurrence of suicidal ideation/death wishes/life weariness in young Sami compared to young majority Swedes was found, but not an increased prevalence of suicide attempts and positive attitudes together with an increased awareness to handle suicide problems could be a contributing factor. Severe circumstances and experience of ethnicity-related bad treatment seems to contribute to increased levels of suicidal plans and attempts in subgroups of Sami.

The Sami is an indigenous people in northern Europe. They inhabit the northern part of Scandinavia and the Kola Peninsula in north-western Russia. Their land has gradually been taken over by the majority people while traditional activities such as reindeer herding, fishing and hunting have declined because of the construction of roads, railways, hydroelectric power-plants and wind-mills, and the rapid increase in the numbers of bears, lynx and wolves. There is a long history of discrimination of the Sami people (1). A recent study found that half of a group of young Swedish Sami reported experiences of ethnicity-related maltreatment by others, and by teachers, which were associated with negative health outcomes (2). Furthermore, the majority of young Sami share the experience of being forced to defend the Sami culture and the Sami way of living. However, the Sami has not experienced to the same extent the poor health, alcohol and drug abuse and suicide as many other indigenous peoples in northern Canada, Alaska, Greenland, and Arctic Russia (3). The life expectancy of the Sami in Scandinavia is the same as that of the majority people and hazardous use of alcohol is at the national mean level or even lower (4–6). There are indications that there is an increased risk of suicide amongst Sami males in Norway, Sweden and Finland, but this increased risk is moderate compared to the majority population (3, 7). There are reports of clusters of suicide in young Sami males in northern Norway and in northern Sweden, but this is not an exclusive problem in the Sami community (8, 9). In general, the suicide rate among young Swedes has not decreased in the way it has amongst those aged 35 and older (10). Research on suicides amongst the Sami is limited and has until now mainly dealt with the Norwegian Sami (2).

In early 2000, there was a cluster of suicides of Sami males aged 20–30 years in a small Sami community (Sameby), and members of this community contacted Umeå University for help. This was to become the beginning of a comprehensive project on the mental health of the reindeer Sami in Sweden (11). This study is part of this project.

The relationship between suicide, culture and socio-economic conditions is not straightforward. Suicide is more prevalent among men, whereas nonfatal suicidal behaviours are more prevalent among women and persons who are young, unmarried, or have a psychiatric disorder (12, 13). A personal history of suicidal behaviour, i.e., suicide ideation, plans and attempts, also contributes to an increased risk if present in significant others (14). There are several ways to understand suicide: as a process starting with the mere idea of suicide as a possibility when life is becoming difficult, and then this can develop into suicidal plans and eventually end up in a suicide attempt and in some cases a completed suicide (15, 16). Another useful perspective is to look at suicide as a psychological accident, which occurs when the demands on an individual suddenly exceeds his/her possibilities to manage the situation (17).

Attitudes and experiences of suicidal expressions in a population could be studied through questionnaires to get an idea of the cultural milieu as regards suicide. Are suicidal experiences rather common in a society, is there closeness to suicide and is there a preparedness to think about suicide as a possibility when life becomes difficult (14, 18–20).

The experience of being discriminated against and ill-treated because of ethnic background might play a role in the background to experiencing life as troublesome and even impossible to cope with.

We wanted to explore suicidal expressions among young Sami compared to Swedes in general, considering:

- The personal history of suicidal experience; and history among significant others
- attitudes toward suicidal problems; and
- the possible role of gender, occupation as a reindeer herder, county of residence, and experience of negative societal treatment.

## Subjects and methods

The study is a cross-sectional investigation into suicide-related issues in a group of young Swedish Sami, 18–28 years of age. The study population consists of young persons with an explicit Sami background. They were identified through different registers or Sami organizations, i.e., the electoral register of the Sami parliament, the Reindeer owner register, Sami Nuorra (the Sami Youth Organization), the Sami Educational Centrum in Jokkmokk, and 19 out of 24 Sami organizations in different parts of Sweden. In 2008, a group of 878 persons were contacted and asked to respond to a postal questionnaire and 516 responded (59% response rate) after one reminder. An absolute majority lived in the most northern part of Sweden (i.e., 241 in Norrbotten, 159 in Västerbotten/Jamtland/Västernorrland, 104 in other counties), and 52 of the participants were reindeer herders. Experience of maltreatment due to ethnicity was very frequent and 269 of the 516 respondents reported this experience. For a further population description, see Omma et al. (2). We have used an age-matched part of a reference group from another study conducted in 2007, investigating health and health-related factors in reindeer herders (21, 22). This reference group consists of a random sample of urban and rural persons living in the northern part of Sweden (where the majority of Swedish Sami are living). Of these 2,000 persons, 69.7% responded. From this original group, we used 105 males and 113 females aged 18–29 years as a reference group who had answered the same questions about suicide behaviour (22).

The two groups are also similar regarding employment and education; 67% of the Sami vs. 63% of the Swedes were working and 33% vs. 37% were studying. However, it was more common to have higher education in the Sami group where 31% had more than 14 years of education compared to 21% in the reference group (p=0.016); in particular, Sami women are better educated (36% vs. 21%) (not shown in the table). Furthermore, 28% of the young Sami live together with their parents compared with 18% in the reference group (p=0.043) (Table I).

**Table I:** Socio-demographic characteristic in Sami (*N*=516) and reference group (*N*=218) aged 18–29 years in %

	Sami	Reference
Gender		
Women	57.2	51.8
Men	42.8	48.2
Missing data	*n*=2	
Years of education		
>9 years	5.6	5.1
10–12 years	63.6	74.3
>14 years	30.7	20.6
Missing data	*n*=2	*n*=4
Family situation		
Alone	23.6	28.8
With partner	31.0	32.6
With partner and child	11.6	10.7
Alone with child	1.2	0.9
With parents	27.9	17.7
Other alternatives	4.7	9.3
Missing data	*n*=7	*n*=4

### Instrument

Parts of the Attitudes toward Suicide (ATTS) Questionnaire were used to cover 4 different aspects of the suicidal process (23).

Contact with suicidal behaviours in significant others: experience of suicide in the family and close persons and if the individual personally knows a person who has committed suicide—“yes” or “no”.

Suicidal expressions: Life weariness (*Have you ever felt that life was not worth living?*), Death wishes (*Have you ever wished you were dead, for instance that you could go to sleep and not wake up?*), Suicidal ideation (*Have you ever thought of taking your life, even if you would not really do it?*), Suicide plans (*Have you ever reached the point where you seriously considered taking your life, or perhaps made plans about how you would go about doing it?*) – “never”, “almost never”, “sometimes” or “often”.

Suicide attempt: Have you ever attempted suicide? “yes” or “no”.

Attitudes toward suicide: *if there is a risk of evoking suicidal thoughts when talking about suicide*, whether *suicide is a topic you should not talk about*, *if it is possible to help a person with suicidal thoughts* and whether *almost everyone at one time has had suicidal thoughts*. Scored from 1 to 5; 1 (“strongly disagree”) to 5 (“strongly agree”).

In the following text, we will use the term suicidal expressions to cover a spectrum of suicidal-related phenomena, including death wishes and suicidal attempts.

Experience of negative societal treatment due to Sami ethnicity was assessed using 3 questions: “Have other people treated you bad because of your Sami background”, “Have you heard teachers saying something bad about the Sami”, and “Has it happened that teachers treated you unfair because of your Sami background”. Response alternatives for the 2 first questions were Yes/No and for the third Yes, Often/Yes, Sometimes/No.

### Statistical analysis

Statistical analyses were performed using the Predictive Analytics Software (PASW), version 18. For group comparisons on own suicidal ideation, plans and suicide attempts responses were dichotomised into 2 response alternatives – never and almost never/sometimes/often (19). On questions about ATTS, a 5-point Likert scale was used 1 (strongly disagree) to 5 (strongly agree) and mean values and SD (standard deviation) were calculated.

The 3 questions referring to maltreatment by teachers or/and others were summarized into the index “Bad treatment” with response alternatives dichotomised to “Yes” (“yes” to at least one of the questions) or “No” (“no” to all of the questions). Multiple logistic regression analysis was conducted to test associations between *suicidal plans* and *attempting suicide* by Bad treatment, Occupation, Gender and Counties in a stepwise forward model.

We only report adjusted odds ratio and their corresponding 95% confidence interval if there are significant differences between assessed variables. Results were considered statistically significant if p<0.05.

### Ethical considerations

This study was approved by the regional research ethics committee in Umeå (§ 06–007) 2007. The questionnaire was voluntary and anonymous. Because of a risk of further stigmatization in studying minority groups, several Sami organizations and especially the Sami Youth organization (Sami Nuorra) have been actively involved in the planning of the project.

## Results

### Contact with suicidal behaviour in significant others

Young Sami reported more experiences of suicide among significant others, 48.6% compared to 38,0% of the reference group (p=0.009). Experiences of suicidal ideation in the family (24.6%) and in others (41.7%) were reported by the Sami compared to 19.5 and 40.8%, respectively among Swedes. There was a tendency for less suicide experiences in the Swedes families. However, this difference was not significant (3.4% vs. 6.1%). Women in both groups reported more experience of suicidal ideation in significant others (Table II).

**Table II:** Suicidal ideations and suicide in family and significant others among young Sami (*N*=508) and reference group (*N*=213) in %

	Sami				Reference				
		
	Men	Women	P/gender	Total	Men	Women	P/gender	Total	P, Sami/reference
Ideation									
In family	19.0	28.8	0.012	24.6	16.7	22.2	ns	19.5	ns
Missing data				*n*=12				*n*=8	
Among others	35.2	46.7	0.011	41.7	30.7	50.0	0.005	40.8	ns
Missing data				*n*=8				*n*=5	
Suicide									
In family	4.7	7.2	ns	6.1	2.0	4.7	ns	3.4	ns
Missing data				*n*=25				*n*=13	
Among others	47.0	49.8	ns	48.6	34.7	41.1	ns	38.0	0.009
Missing data				*n*=12				*n*=5	

### Suicidal expressions

Suicidal expressions were common among young Sami and Swedes but the Sami reported a higher prevalence of *Life weariness* (63% vs. 50%, p=0.002)*, death wishes* (49% vs. 37%, p=0.002) and *suicidal ideation* (45% vs. 37%, p=0.042). There were gender differences in both groups. Sami women reported more life weariness, death wishes, suicidal ideation and suicide plans compared to Sami men. In the reference group, women reported more life weariness and death wishes compared to men. A fifth of both groups reported having had suicide plans (no significant difference) (Table III).

**Table III:** Suicidal expressions in young Sami (*N*=516) and reference group (*N*=218) in %

	Primary response alternatives								Merged response alternatives						
		
	Never		Almost never		Sometimes		Often		Almost never/sometimes/often						
					
	Sami	Reference	Sami	Reference	Sami	Reference	Sami	Reference	Sami	P, Sami/gender	Reference	P, Reference/gender	Sami total	Ref. total	P, Sami/reference
Life weariness															
Men	49.5	58.8	34.1	23.5	13.2	13.7	3.2	3.9	50,5	0.000	41.2	0.010	62.5	50.0	0.002
Women	28.5	42.0	41.4	34.8	24.7	17.0	5.4	6.3	71.5		58.0				
Death wishes															
Men	62.4	70.6	23.4	14.7	12.8	13.7	1.4	1.0	37.6	0.000	29.4	0.025	49.4	36.6	0.002
Women	41.8	56.8	34.7	27.0	19.4	10.8	4.1	5.4	58.2		43.2				
Suicidal ideation															
Men	65.1	68.6	22.0	16.7	10.6	13.7	2.3	1.0	34.9	0.000	31.4	ns	45.1	36.9	0.042
Women	47.3	58.0	32.0	25.0	16.7	11.6	4.1	5.4	52.7		42.0				
Suicide plans															
Men	85.8	84.2	8.2	9.9	5.5	5.0	0.5	1.0	14.2	0.012	15.8	ns	18.9	18.3	ns
Women	77.6	79.5	14.6	12.5	6.1	4.5	3.6	22.4			20.5				

The most severe suicidal behaviour, *suicide attempt*, shows an opposite tendency with higher prevalence of suicide attempts in the reference group, 7, 9% compared to 5, 5% of the Sami, although the difference is not statistically significant. A similar tendency of higher prevalence of suicide attempts among women compared to men was reported in both groups: Sami (women 7.1%, men 3.2%) and Swedes (women 10.7%, men 4.9%).

### Suicidal expression in subgroups of Sami

The reindeer herders and those being badly treated due to ethnicity reported a higher degree of *suicide attempts* and of having had *plans to take own life* compared to Sami without this experience. Similarly, more Sami women and those living in Västerbotten/Jamtland/Västernorrland reported having had plans to take their own life compared to Sami men and those living in other counties. The odds ratios and 95% confidence intervals are shown in Tables IV and V.

**Table IV:** Suicide attempts and plans to take own life by occupation (to be a reindeer herder) bad treatment, gender and counties in 18–28 years old Sami in Sweden (*N*=494–514) in %

	Have you ever attempted suicide?			Have you ever planned to take own life?		
		
	Yes	No	P, Yes/no	Never	Almost never/sometimes/often	P, Never/almost never, sometimes, often
Bad treatment^1^						
Yes	7.3	92.7	0.031	74.9	25.1	0.000
No	3.0	97.0		87.7	12.3	
Reindeer herder^2^						
Yes	11.8	88.2	0.037	69.2	30.8	0.021
No	4.8	95.2		82.5	17.5	
Gender^3^						
Men	3.2	96.8	0.054	85.8	14.2	0.019
Women	7.1	92.9		77.6	22.4	
Counties^4^						
Norrbotten	4.1	95.9	ns	85.4	14.6	0.014
Vasterbotten/Jamtland/Vasternorrland	7.5	92.5		73.9	26.1	
Other Counties	4.8	95.2		82.7	17.3	

1–4 From any other counties in Sweden (except the four indicated with superscript number in this table).

**Table V:** Unadjusted and adjusted odds ratios for suicide attempts and plans to take own life with bad treatment due to ethnicity, reindeer herder, gender and counties as explaining variables.

	Suicide attempts		Plans to take own life	
		
	uOR (95% CI)	aOR (95% CI)^1^	uOR (95% CI)	aOR (95% CI)
Bad treatment due to ethnicity				
No	1	1	1	1
Yes	2.40 (1.04–5.55)	ns	2.28 (1.43–3.64)	2.32 (1.41–3.81)
Reindeer herder				
No	1	1	1	
Yes	2.67 (1.03–6.92)	3.34 (1.13–9.86)	2.09 (1.11–3.95)	2.11 (1.02–4.37)
Gender				
Men	1	1	1	
Women	ns	3.41 (1.29–9.00)	1.75 (1.09–2.79)	1.86 (1.12–3.08)
Counties				
Norrbotten	1	1	1	1
Vasterbotten/Vasternorrland/Jamtland	ns	ns	2.07 (1.25–3.42)	2.22 (1.32–3.78)
Other counties	ns	ns	ns	ns

ns = not significant, uOR = unadjusted odds ratio, aOR = adjusted odds ratio.

1 Regression analyses method stepwise forward.

### Attitudes to suicide

There were differences regarding attitudes to suicide in the 2 populations. The Sami were more likely to disagree that talking about suicide increased the *risk of evoking suicidal thoughts*. The Sami were more likely to agree with the *possibility to help people with suicidal problems* and that everyone *has had suicidal thoughts*. In the Sami group, women disagreed more strongly to “you should not talk about suicide”. In the reference group, women more strongly disagreed with the “risk of evoking suicidal thoughts when talking about them” (Table VI).

**Table VI:** Attitudes to suicide in young Sami (n=505–508) and reference group, (n=212–214) gender specific and total

	Sami				Reference				p
			
	Men Mean (SD)	Women Mean (SD)	P Sami/gender	Total Mean (SD)	Men Mean (SD)	Women Mean (SD)	P Reference/gender	Total Mean (SD)	Sami/reference
Risk-evoking suicidal thoughts	2.10 (1.02)	2.09 (0.93)	ns	2.10 (0.973)	2.40 (1.02)	2.09 (0.94)	0.004	2.26 (0.97)	0.041
Not talk about suicide	2.15 (1.19)	1.71 (0.98)	0.000	1.90 (1.20)	2.17 (.17)	1.75 (0.94)	ns	1.95 (1.07)	ns
Always possible to help	4.12 (0.91)	3.98 (0.83)	ns	4.04 (0.87)	3.72 (1.12)	3.82 (1.02)	ns	3.77 (1.07)	0.000
Almost everyone has had suicidal thoughts	2.78 (1.25)	2.81 (1.16)	ns	2.80 (1.10)	2.37 (1.24)	2.64 (1.24)	ns	2.51 (1.25)	0.004

Scoring 1 (strongly disagree) to 5 (strongly agree).

## Discussion

The current study found that young Sami experienced a higher prevalence of suicidal expressions and suicide in significant others but not a higher prevalence of suicide attempts compared to young Swedes. Reindeer herders, Sami with experience of ethnicity-related maltreatment, those living in Västerbotten/Jamtland/Västernorrland and Sami women reported a markedly higher prevalence of suicide plans. Likewise reindeer herders and women had a about 3-fold higher odds of attempting suicide. A more positive attitude toward possibilities to act against suicidal problems was indicated in the Sami group.

The representativeness of the sample requires some explanation. The young Sami were found through different registers and by their connections to Sami organizations. The identity is based on self-identification (24). As no ethnic registers are allowed in Sweden and most Sami are assimilated into the Swedish society, this is the only way to locate Sami participants in a study.

The response rate (59%) is deemed satisfactory considering the problem of finding young persons who in many cases have not settled down. Under these circumstances, the sample is deemed representative for young persons with an explicit Sami identity. ATTS is used to indicate factors that contribute to the understanding of suicide and suicidal behaviour, but is not expected to illuminate the entire phenomenon. We have used a selection of attitude items from ATTS, as the intention was to keep the number of items low.

The Sami is a small indigenous group with strong family relations and coherence, which means that as soon as a suicide occurs many will be affected. This could be a reason for the higher prevalence of young Sami reporting completed suicide in significant others. A number of studies have shown an association between the experience of suicidal behaviour in significant others and own suicidal behaviour which thus constitute an important risk factor for suicidal behaviour (25).

It is interesting to see that there is a substantial number of missing data in both groups regarding suicidal problems in the family. This might indicate that the issue is sensitive or that the respondents are uncertain and do not know the answer.

A high proportion of both Sami and Swedes reported own suicidal expressions. However, the Sami more frequently reported life weariness, death wishes and suicidal ideation. This might be seen as part of an existential complex which can have different sources, for example an individual's desire to contribute to saving the Sami language and culture from extinction and a response to external attacks on Sami culture as an obstacle to social development (1).

Experience of racial discrimination is consistently found to be associated with a range of negative mental health measures (26, 27). This is supported by findings in a recent study by Omma et al. (28) where young Sami experiencing ethnicity-related maltreatment by others were less prone to feel calm and more prone to have worries. Furthermore, those who had perceived ethnicity-related maltreatment by teacher more often felt sad and depressed.

One possible way to understand these elevated risks is the severe circumstances and existential demands that these young Sami are facing. Reindeer herding is under pressure in many ways, especially in the southern part of the Sapmi. There are severe and persistent conflicts about land use and the Sami have lost herding areas. Therefore, the Sami is suffering from both a historical loss of land, which can mean a negative impact on moods and mental health (29) and an on-going loss of land (30). Other distressing factors could be the low economic standards of the reindeer herders because of the strained conditions. However, herding is merely seen as a way of living and not as an occupation, and those engaged often feel it important to continue despite the hardships (20, 21). According to William's and Pollack, suicide can be conceptualized as a “cry of pain”, the response to a situation that has 3 components: defeat, no escape and no rescue. If the possibility to escape (e.g., from painful self-perception) is arrested and/or there is no hope about rescue, the situation can be impossible to bear (31, 32). However, the concept of suicide as an “Act of Pain” as opposed to a “Cry of pain” seems more reasonable. As there is no indication of an elevated prevalence of mental illness or drinking problems in this Sami population (4, 28), we believe there is an accumulation of distressing, negative circumstances that really could be life threatening especially for the reindeer herders.

The Sami women reported more suicide attempts and plans, and women in both groups reported more suicidal expressions than men. Women, especially in Western societies, report a higher occurrence of depression (33, 34), suicidal thoughts and plans, and they also report more suicidal attempts than men, whilst men in Western societies more often die from completed suicides (13). This gender differences are sometimes discussed as a gender paradox. One possible explanation is a higher and perhaps genetically caused vulnerability in women, but also perhaps due to the effect of socio-culturally determined gender roles. Another possibility is differences in affective/cognitive style, with men more likely to act out. Women perceive and reflect about feelings, thoughts and experience before communicating them. This is perhaps a protective strategy, which helps to understand and regulate feelings and behaviour and which might increase the possibilities to handle negative experiences and might even function as a protective force against completed suicide. This is in contrast to men, who maybe do not allow themselves to think about this issue and to communicate with others. The man's tendency to act instead of communicating perceived problems and threats might make them more vulnerable to active suicide behaviour.

There has been a concern for several years about suicide-related problems and this has been highlighted in meetings amongst different groups and organizations of Sami. This might have had an impact on attitudes to suicide and contributed to the finding that even though the Sami youth report a higher experience of suicide in significant others and higher prevalence of own suicidal expressions, they report no more plans and attempts than the majority group. In a qualitative study, the author found that suicide is a common topic amongst young Sami, making them more aware of the problem and the need to reduce the suicidal risk in the group (35).

Silviken et al. found an increased risk of suicide mortality in a Norwegian Sami cohort compared to a reference population in the study period 1970–1998 (9). In a follow up study, no ethnic differences of suicide attempts were found between Sami adolescents (mean age 16.9) and their non-Sami peers (8). The prevalence of suicide attempts was 10.5% in the Norwegian Sami adolescent population compared to 5.5% in this study. The prevalence of suicide ideation was 19% for women and 14% for men in this population, compared to 53% and 35%, respectively in the present study (36). Differences in age, time for the assessment, and the posing of questions make comparisons difficult between studies.

In the SLiCA project (the Survey of Living Conditions in the Arctic) amongst indigenous people age 15–34 years, suicidal thoughts were present in 5% (men) and 12% (women) among Swedish Sami, and 9% (men) and 31% (women) among Norwegian Sami (37). Those proportions are low compared to the findings in this study and could be due to methodological differences. The SLiCA study used face-to-face interviews.

We found an increased awareness of the suicide problem in the Sami community and there are on-going attempts to deal with this. For example, the SSR (the Swedish Sami organization) is organizing seminars on lifestyle issues with young male Sami. We have been invited to discuss and inform about research results at several meetings with different Sami groups who consider mental health in the Sami population to be important.

It is important that problems for the Sami culture are addressed by the majority community. The need for culturally adapted health care for the Sami should be met and the issue of discrimination and negative treatment of the Sami should be addressed. Suicidal behaviour is not necessarily primarily an expression of a wish to die, but part of an internal existential dialogue contributing to building a human identity. It is thus important not only to consider different risk factors for suicidal behaviour, but also to discuss possible protective factors. Strong family ties and a positive ethnic group identity might be of particular importance for this group.
